# Particulars of the Awards for 1889

**Published:** 1889-08-03

**Authors:** 


### PARTICULARS OF THE AWARDS FOR 1889.

### TWENTY-TWO GENERAL HOSPITALS.

Charing Cross, West Strand, W.C., £989 11s. 8d.; French, Lisle-street, W.C., £208 6s. 8d.; German, Dalston-lane, E., £677 1s. 8d.; Great Northern Central, Caledonian-road, N., £312 10s.; Guy's, £208 6s. 8d.; Italian, Queen-square, W.C., £72 18s. 4d.; King's College, Lincoln's-inn-fields, W.C., £1,406 5s.; London, Whitechapel, E., £3,333 6s. 8d.; Metropolitan, Kingsland-road, N., £260 8s. 4d.; Miller Hospital and Royal Kent Dispensary, £281 5s.; North-West London, Kentish-town-road, N.W., £322 18s. 4d.; Poplar, East India-road, E., £218 15s.; Royal Free, Gray's-inn-road, W.C., £958 6s. 8d.; St. George's, Hyde Park Corner, S.W., £1,614 11s. 8d.; SS. John and Elizabeth, Great Ormond-street, W.C., £125; St. Mary's, Paddington, W., £1,875; Seamen's, Greenwich, S.E., £677 1s. 8d.; The Middlesex, Charles-street, W., £2,031 5s.; Tottenham Training Hospital, Tottenham, N., £281 5s.; University College, Gower-street, W.C., £1,302 1s. 8d.; West London, Hammersmith, W., £520 16s. 8d.; Westminster, Broad-sanctuary, S.W., £1,093 15s.

### SPECIAL HOSPITALS.

FIVE CHEST HOSPITALS.—City of London Hospital for Diseases of the Chest, Victoria Park, E., £937 10s.; Hospital for Consumption, Brompton, S.W., £1,718 15s.; North London Consumption Hospital, Hampstead, £364 11s. 8d.; Royal Hospital for Diseases of the Chest, City-road, £385 8s. 4d.; Royal National Hospital for Consumption (Ventnor), £208 6s. 8d.

TWELVE CHILDREN'S HOSPITALS.—Alexandra, Queen-square, W.C., £208 6s. 8d.; Belgrave, 1, Cumberland-street, Pimlico, S.W., £140 12s. 6d.; Cheyne Hospital for Children, Chelsea, S.W., £109 7s. 6d.; East London, Shadwell, E., £416 13s. 4d.; Evelina, Southwark-bridge-road, S.E., £468 15s.; Home for Children, Maida-vale, N.W., £83 6s. 8d.; Home for Sick Children and South London Dispensary for Women, Sydenham, S.E., £145 16s. 8d.; Hospital for Sick Children, Great Ormond-street, W.C., £781 5s.; North Eastern, 327, Hackney-road, E., £322 18s. 4d.; Paddington Green Hospital for Sick Children, £161 9s. 2d.; Victoria, Queen's-road, Chelsea, S.W., £354 3s. 4d.; "The Vine," Sevenoaks, £31 5s.

THREE LYING-IN HOSPITALS.—British, Endell-street, W.C., £62 10s.; General, York-road, Lambeth, S.E., £88 10s. 10d.; Queen Charlotte's, Marylebone-road, W., £312 10s.

SIX HOSPITALS FOR WOMEN.—Chelsea Hospital for Women, Fulham-road, S.W., £197 18s. 4d.; Hospital for Women, Soho-square, W., £416 13s. 4d.; Grosvenor Hospital for Women and Children, Vincent-square, S.W., £83 6s. 8d.; New Hospital for Women, 322, Marylebone-road, W., £130 4s. 2d.; Royal Hospital for Diseases of Children and Women, Waterloo-bridge-road, S.E., £229 3s. 4d.,; Samaritan Free, Lower Seymour-street, W., £479 3s. 4d.

### TWENTY-SEVEN OTHER SPECIAL HOSPITALS.

Cancer, Brompton, S.W., £520 16s. 8d.; London Fever, Liverpool-road, N., £520 16s. 8d.; Gordon Hospital for Fistula, £20 16s. 8d.; St. Mark's (Fistula), City-road, E.C., £104 3s. 4d.; National, for Diseases of the Heart, Soho-square, W., £140 12s. 6d.; Female Lock, Westbourne Green, W., £260 8s. 4d.; Male Lock, Dean-street, Soho, W., £62 10s.; Hospital for Epilepsy, Paralysis, and other Diseases of the Nervous System, Portland-terrace, N.W., £104 3s. 4d.; National, for the Paralysed and Epileptic, Queen-square, W.C., £531 5s.; *West End Hospital for Diseases of the Nervous System, nil;* Central London Ophthalmic, Gray's-inn-road, W.C., £67 14s. 2d.; Royal London Ophthalmic, Moorfields, E.C., £572 18s. 4d.; Royal South London Ophthalmic, St. George's-circus, S.E., £93 15s.; Royal Westminster Ophthalmic, King William-street, W.C., £104 3s. 4d.; Western Ophthalmic, Marylebone-road, W., £30 4s. 2d.; City Orthopædic, Hatton-garden, E.C., £78 2s. 6d.; National Orthopædic, Great Portland-street, W., £31 5s.; Royal Orthopædic, Hanover-square, W., £93 15s.; Hospital for Diseases of the Skin, Stamford-street, S.E., £26 0s. 10d.; *St. John's, for Diseases of the Skin, Leicester-square, W.C., nil*; Central London Throat and Ear, Gray's-inn-road, W.C., £57 5s. 10d.; Royal Ear Hospital, £10 8s. 4d.; Hospital for Diseases of the Throat, Golden-square, W., £20 16s. 8d.; London Homœopathic, Great Ormond-street, W.C., £250; London Temperance, Hampstead-road, N.W., £416 13s. 4d.; The Dental, Leicester-square, W.C., £93 15s.; National Dental, 149, Great Portland-street, W., £20 16s. 8d.

### TWENTY CONVALESCENT HOSPITALS.

Metropolitan Convalescent Institution, Walton-on-Thames, &c., £677 1s. 8d.; Bexhill-on-Sea Convalescent Home, £260 8s. 4d.; All Saints' Convalescent Home, Eastbourne, £572 18s. 4d.; Mrs. Gladstone's Convalescent Home, Woodford, E., £177 1s. 8d.; Hanwell Convalescent Home, Middlesex, £26 0s. 10d.; Herbert Convalescent Hospital, Bournemouth, £26 0s. 10d.; King's College Convalescent Hospital, Hemel Hempstead, £130 4s. 2d.; Mrs. Kitto's Convalescent Hospital, Reigate, Surrey, £78 2s. 6d.; Mrs. Marshman's Convalescent Hospital, Brighton, £83 6s. 8d.; Mary Wardell Convalescent Home, Great Stanmore, £125; Morley Convalescent Hospital, St. Margaret's, Kent, £57 5s. 10d.; Princess Frederica's Convalescent Home, East Molesey, £36 9s. 2d.; Children's Convalescent Homes, Ramsgate, £26 0s. 10d.; St. Andrew's Convalescent Homes, Clewer, £145 16s. 8d.; St. Andrew's Convalescent Homes, Folkestone, £145 16s. 8d.; St. Barnabas Convalescent Home, Ramsgate, £15 12s. 6d.; St. Leonards-on-Sea Convalescent Hospital for Children, £104 3s. 4d.; St. Mary Magdalene Convalescent Home, Paddington, W., £52 1s. 8d.; St. Michael's Convalescent Home, Westgate-on-Sea, £36 9s. 2d.; Seaside Convalescent Hospital, Seaford, £104 3s. 4d.

### ELEVEN COTTAGE HOSPITALS.

Beckenham, £46 17s. 6d.; Blackheath and Charlton, £67 14s. 2d.; Burstead, £20 16s. 8d.; Chislehurst, &c., £52 1s. 8d.; Eltham, £31 5s.; Enfield, £31 5s.; Epsom and Ewell, £41 13s. 4d.; Reigate and Redhill, £72 18s. 4d.; Shedfield, £10 8s. 4d.; Sidcup, £26 0s. 10d.; Wimbledon, £36 9s. 2d.

### SEVEN INSTITUTIONS.

Establishment for Gentlewomen, Harley-street, W., £83 6s. 8d.; Hampstead Home Hospital, £67 14s. 2d.; National Sanatorium, Bournemouth, £72 18s. 4d.; Royal Sea Bathing Infirmary, Margate, £572 18s. 4d.; Invalid Asylum, Stoke Newington, £72 18s. 4d.; "Firs Home," Bournemouth, £26 0s. 10d.; "St. Catherine's Home," Ventnor, £48 19s. 2d.

### FIFTY DISPENSARIES.

Battersea (Provident), £57 5s. 10d.; Brixton, &c., £46 17s. 6d.; Brompton (Provident), £22 18s. 4d.; Camberwell (Provident), £98 19s. 2d.; Chelsea, £46 17s. 6d.; Child's Hill (Provident), £12 10s.; City, £119 15s. 10d.; City of London and East London, £22 18s. 4d.; Clapham (General), £36 9s. 2d.; Eastern, £31 5s.; Farringdon, £52 1s. 8d.; Finsbury, £57 5s. 10d.; Forest Hill, £36 9s. 2d.; Gipsy Hill (Provident), £20 16s. 8d.; Hampstead (Provident), £52 1s. 8d.; Holloway and North Islington, £78 2s. 6d.; Islington, £62 10s.; Kensington, £83 6s. 8d.; Kilburn, &c., £52 1s. 8d.; Kilburn (Provident), £31 5s.; London, Spitalfields, E., £17 14s. 2d.; Margaret-street Infirmary for Consumption, W.,

£15 12s. 6d.; Metropolitan (Fore-street, E.C.), £41 13s. 4d.; Notting Hill (Provident), £26 0s. 10d.; Paddington (Provident), £41 13s. 4d.; Portland Town (Free), £20 16s. 8d.; Portobello-road (Provident), £5 4s. 2d.; Public, Stanhope-street, W.C., £57 5s. 10d.; Queen Adelaide's, Bethnal Green, E., £46 17s. 6d.; Royal General, Bartholomew-close, E.C., £57 5s. 10d.; Royal Maternity Charity, Finsbury-square, E.C., £125; Royal Pimlico (Provident), £52 1s. 8d.; Royal South London, St. George's-circus, S.E., £62 10s.; SS. George's and James's, King's-street, W., £67 14s. 2d.; St. George's, Hanover-square (Provident), £52 1s. 8d.; St. John's Wood (Provident), £23 19s. 2d.; St. Marylebone (General), £41 13s. 4d.; St. Pancras and Northern, £37 10s.; SS. Paul and Barnabas (Provident), Pimlico, S.W., £52 1s. 8d.; South Lambeth, £57 5s. 10d.; South London Medical Aid Institute, Waterloo-bridge-road, S.E., £31 5s.; Stamford-hill, &c., Stoke Newington, N., £54 3s. 4d.; Tower Hamlets, Stepney, E., £46 17s. 6d.; Wandsworth Common (Provident), £10 8s. 4d.; Westbourne (Provident), £20 16s. 8d.; West Dulwich (Provident), £7 5s. 10d.; Western, Rochester-row, Westminster, S.W., £46 17s. 6d.; Western General, Marylebone-road, S.W., £135 8s. 4d.; West Ham and Stratford, E., £62 10s.; Westminster General, Gerrard-street, W., £36 9s. 2d.

